# Weight loss outcomes are generally worse for dogs and cats with class II obesity, defined as > 40% overweight

**DOI:** 10.1038/s41598-023-50197-y

**Published:** 2023-12-27

**Authors:** H. A. O. Broome, G. R. T. Woods-Lee, J. Flanagan, V. Biourge, A. J. German

**Affiliations:** 1 Institute of Infection, Veterinary and Ecological Sciences, University of Liverpool, Liverpool, UK; 2 Institute of Life Course and Medical Sciences, University of Liverpool, Liverpool, UK; 3 Royal Canin Research Centre, Aimargues, France

**Keywords:** Animal physiology, Obesity

## Abstract

In pet dogs and cats, adiposity is most-often estimated clinically using a 9-category body condition score (BCS), with BCS 9 equating to ~ 40% overweight. Animals that are more overweight (> 40%) are seen in clinical practice but are not appropriately depicted by descriptions in the existing categories. To determine whether being > 40% overweight has clinical relevance, this study aimed to compare the outcomes of weight management in animals that were > 40% overweight with those < 40% overweight. Records of dogs and cats attending a specialist obesity care clinic, where adiposity is determined using dual-energy X-ray absorptiometry (DXA), were reviewed. Animals were assigned to two classes (class I ≤ 40% overweight: 118/398 [40%] dogs and 68/116 [59%] cats; class II, > 40% overweight: 180/398 [60%] dogs and 48/116 [41%] cats) based on DXA results, and weight loss outcomes were compared. Fewer class II dogs obesity completed weight management than class I dogs (*P* < 0.001), rate of weight loss was also slower (*P* = 0.012) and lean tissue loss greater (*P* < 0.001). Compared with class I, cats with class II obesity lost more weight (*P* = 0.048) albeit over a longer period (*P* = 0.043) leading to greater lean tissue loss (*P* = 0.004). Approximately half the pets presenting to a specialist clinic were have class II obesity (> 40% overweight), and some weight loss outcomes are worse for these animals.

## Introduction

Obesity is defined as “a disease in which excess body fat has accumulated such that health may be adversely affected”^1^ and, in dogs and cats, it can adversely affect lifespan^2^ and quality of life^3,4^, and also increase the risk of comorbidities^5–10^. As part of the diagnostic process, the amount of body fat (adiposity) can be precisely quantified using a tool such as dual-energy X-ray absorptiometry (DXA), which is suggested to be the gold-standard method^11,12^ but is limited to research institutions specialist veterinary practice^13–16^. Instead, subjective clinical methods for estimating body fat percentage can be used, such as the body fat index and morphometry^17,18^. However, the most widely-accepted clinical approach by veterinary professionals is to assess body condition score (BCS), which correlates well with body fat mass, and can be used to estimate how overweight a dog or cat is^19,20^. Of the systems available, a 9-category system is generally recommended, whereby, with each category between 5 and 9 equating to around 10% excess weight, and a score of 9 corresponding to an animal being approximately 40% overweight^19,20^. However, two of the study authors (GW, AG) have observed many dogs and cats in their clinical practice whose characteristics do not match with (i.e. exceed) the description for score 9; DXA measurements in such patients suggest that they might be > 40% overweight (GW & AG, personal observations).

Body mass index (BMI) is a common clinical measure for determining adiposity in humans, and individuals with excess adiposity were originally classified as overweight (BMI 25–30) or obese (BMI > 30). More recently, to reflect degrees of adiposity better, three classes of obesity have been recognised (class I, BMI of 30 to < 35; class II, BMI 35 and < 40;class III, BMI > 40)^21^. This subclassification is somewhat arbitrary but is justified by the fact that health outcomes (e.g. morbidity and mortality risk) differ amongst classes^22^, with the greatest risks for individuals with class III obesity^23–27^. Adopting a similar approach might be useful in dogs and cats. Currently, both overweight and obese categories have been defined based on BCS, and there is epidemiological evidence to suggest differences in the comorbidities seen between these categories^8,9^. However, to date, there has been no attempt to define obesity further into different classes in dogs and cats as in humans. Given the widespread use of the 9-category BCS, animals could be defined as class I or class II obesity, separated by a cut-point of 40% overweight (the current limit of the 9-point BCS). Such an approach could help veterinary professionals if clinically-relevant differences exist between animals in different classes; examples of such differences include differences in adverse health consequences (e.g. risk of comorbidities) or differences in clinical outcomes of weight reduction (e.g. likelihood of reaching target weight, amount of lean tissue loss etc.).

For the last 18 years, we have been running a specialist obesity care clinic for dogs and cats, and routinely use body composition data measured by DXA both in our clinical assessment before (e.g. to determine adiposity and calculate ideal weight) and after (e.g. to quantify changes in body fat and lean tissue mass) weight reduction^13–16^. Our first aim was to use these data to assign animals to two obesity classes (class I and II), and then to compare weight reduction outcomes between them, including (but not limited to) percentage weight loss, rate of weight loss, energy intake during weight loss and change in body composition. A second aim was to determine whether the prevalence of these two obesity classes had changed over time.

## Results

### Study animals

A total of 361 dogs and 135 cats were seen by the *specialist obesity care clinic* between 2004 and 2022. After the removal of animals whose weight reduction programmes were ongoing and those whose initial body composition was not measured by DXA, 298 dogs and 116 cats remained eligible for the study. There were 67 different dog breeds represented (Table 1), of which Labrador retrievers (68, 22%), mixed breed dogs (42, 14%), Cavalier King Charles Spaniels (24, 8%), Golden Retrievers (15, 5%) and pugs (12, 4%), were most common. There were 157 male dogs (53%, 146 neutered) and 141 female dogs (47%, 130 neutered), whilst the median age at enrolment was 78 months (7 to 163 months). Of the 116 cats studied (Table 2), the majority were of the Domestic shorthair breed (104, 90%). The rest of the population comprised British short hairs (3), Siamese (2), Domestic long-hair (3), one Maine Coon (1), one Selkirk rex (1), one Burmese (1) and one Bengal cross (1). There were 64 male (55%) and 52 female (45%) cats, all neutered, and the median age at enrolment was 87 months (15 to 178 months).

**Table 1:** Baseline variables in dogs with class I and class II obesity.

Variable	Obesity class^1^		*P* value^2^	Effect size^3^		
	I	II		Effect size	95% CI^4^	Interpretation
Number	118 (40%)	180 (60%)	–	–	–	–
Age (months)	82 (16–156)	74.5 (7–163)	0.754	0.05	− 0.09, 0.18	Very small
Breed	American Bulldog (1) Basset Hound (1) Beagle (1) Bichon Frise (3) Border Collie (1) Boxer (1) Bull dog (4) Cavalier King Charles Spaniel (5) Cocker Spaniel (2) Cross Breed (17) Doberman (3) English Bull Terrier (1) Field Spaniel (1) Flat Coated Retriever (1) French Bull Dog (1) German Shephard (1) Golden Retriever (3) Iris Setter (1) Labrador (40) Lancashire Heeler (1) Lhasa Apso (2) Miniature Poodle (1) Miniature Schnauzer (3) Newfoundland (2) Old English Sheep Dog (1) Pug (5) Rottweiler (5) Shih Tzu (1) Siberian Huskey (1) Springer Spaniel (1) Staffordshire Bull Terrier (2) Yorkshire Terrier (5)	Affenpinscher (1) Alaskan Malamute (2) American Bulldog (2) Beagle (2) Bernese Mountain Dog (2) Bichon Frise (2) Border Collie (9) Border Terrier (1) Boxer (1) Bull Mastiff (1) Bull dog (4) Cairn Terrier (2) Cavalier King Charles Spaniel (19) Chihuahua (3) Cocker Spaniel (5) Corgi (1) Cross Breed (25) Dachshund (7) Doberman (1) English Bull Terrier (1) Field Spaniel (1) French Bull Dog (1) German Shephard (2) Glen of Imaal Terrier (1) Golden Retriever (12) Jack Russell (5) Japanese Akita (2) Labrador (28) Lhasa Apso (2) Newfoundland (2) Norfolk Terrier (1) Norwegian Elk Hound (1) Patterdale Terrier (1) Pointer (1) Poland Lowland Sheep Dog (1) Poodle (1) Pug (7) Rhodesian Ridgeback (1) Rottweiler (1) Rough Collie (2) Samoyed (1) Schiperke (1) Scottish Terrier (1) Shih Tzu (2) Siberian Huskey (1) Springer Spaniel (3) Staffordshire Bull Terrier (2) Tibetan Terrier (1) Weimaraner (1) Yorkshire Terrier (3)	–	–	–	
Sex						
Male (intact)	6 (2%)	5 (2%)	Sex:			
Male (neutered)	55 (18%)	91 (31%)	0.860	0.00	0.00, 1.00	Small
Female (intact)	3 (1%)	8 (3%)	Neuter:			
Female (neutered)	54 (18%)	76 (26%)	0.884	0.00	1.0, 1.00	Small
Comorbidities	Respiratory (14) Cardiac (9) Gastrointestinal (12) Renal (2) Urinary (4) Immunological (2) Dermatological (24) Endocrinological (3) Dental/oral (5) Orthopaedic (47) Neurological (4) Ocular (8) Other (6)	Respiratory (19) Cardiac (11) Gastrointestinal (8) Renal (1) Urinary (4) Immunological (0) Dermatological (45) Endocrinological (12) Dental/oral (8) Orthopaedic (68) Neurological (10) Ocular (12) Other (5)	–	**–**	**–**	
Any comorbidity	103 (87%)	149 (83%)	0.754	0.02	0.00, 1.00	Small
Orthopaedic disease	47 (40%)	68 (38%)	0.794	0.00	0.00, 1.00	Small
Cardiorespiratory disease	18 (15%)	22 (12%)	0.754	0.00	0.00, 1.00	Small
Dermatological disease	24 (20%)	45 (25%)	0.754	0.00	0.00, 1.00	Small
Body fat percentage^5^	39.8 (27.3–50.7)	48.0 (33.3–63.2)	** < 0.001**	**– 0.69**	** − **0.75, − 0.61	Very large
Percentage overweight	28 (11–40)	56 (40–191)	** < 0.001**	** − 1.00**	** − **1.00, − 1.00	Very large
Diet used^6^						
HPMFdry	22	20	Dry vs. wet vs. mix:			
HPMF wet	2	5	0.754	0.00	0.00, 1.00	Small
HPMF dry and wet	1	2	HPMF vs. HPHF:			
HPHF dry	73	116	0.754	0.00	0.00, 1.00	Small
HPHF dry and HPMF wet	2	3				
HPHF dry and wet	18	34				

^1^Class I and II obesity defined as < 40% and > 40% overweight, based on analysis of body composition. ^2^Benjamini-Hochberg-adjusted P-values^68^ reported for categorical data are from either Chi squared or Fisher’s exact test; those for continuous data are from Mann Whitney tests. ^3^Effect size reported was Cohen’s V for tests involving categorical data and rank biserial for continuous data. The magnitude of the effect size is reported according to the rules of Cohen^69^ for Cramer’s V and Funder and Ozer^70^ for the rank biserial. ^4^95% CI 95% confidence interval. ^5^Body fat percentage determined by dual-energy X-ray absorptiometry. ^6^Ideal weight determined from body composition analysis from DXA, as described in the “Methods” Section. ^7^*HPMF* high protein medium fibre, *HPHF* high protein high fibre.

Significant values are in bold.

**Table 2:** Baseline variables in cats with class I and class II obesity.

Variable	Obesity class^1^		*P* value^2^	Effect size^3^		
	I	II		Effect size	95% CI^4^	Interpretation
Number	68 (59%)	48 (41%)	–	–	–	–
Age (months)	87 (15–178)	96 (25–156)	0.435	− 0.14	− 0.34, 0.08	Small
Breed	Burmese (1) British Short Hair (2) Domestic Short Hair (149) Domestic Long Hair (3) Selkirk Rex (1) Siamese (1)	British Short Hair (1) Bengal Cross (1) Domestic Short Hair (45) Main Coon (1) Siamese (1)	–	–	–	
Sex						
Male neutered	44 (38%)	20 (17%)	**0.037**	0.21	0.00, 1.00	Small
Female neutered	24 (21%)	28 (24%)				
Comorbidities	Respiratory (2) Cardiac (7) Gastrointestinal (2) Renal (3) Urinary (8) Dermatological (7) Endocrinological (4) Dental/oral (5) Orthopaedic (3) Neurological (0) Ocular (0)	Respiratory (2) Cardiac (4) Gastrointestinal (3) Renal (1) Urinary (6) Dermatological (1) Endocrinological (2) Dental/oral (5) Orthopaedic (1) Neurological (1) Ocular (1)	–	–	–	
Any comorbidity	31 (46%)	22 (45%)	0.979	0.00	0.00, 1.00	Small
Cardiorespiratory disease	9 (13%)	6 (13%)	0.979	0.00	0.00, 1.00	Small
Body fat percentage^5^	31.0 (17.3–44.6)	42.0 (34.9–54.7)	** < 0.001**	** − 0.93**	** − **0.95, − 0.89	Very large
Percentage overweight^6^	26 (6–40)	58 (41–133)	** < 0.001**	** − 1.00**	** − **1.00, −1.00	Very large
Diet used^7^						
HPMF dry	12	6	Dry vs. wet vs. mix:			
HPMF wet	1	0	0.512	0.09	0.00, 1.00	Small
HPMF dry and wet	11	5	HPMF vs. HPHF:			
HPHF dry	19	15	0.784	0.00	0.00, 1.00	Small
HPHF wet	0	1				
HPMF and HPHF dry	1	0				
HPHF dry and HPMF wet	20	16				
HPHF dry, HPMF wet and HPHF wet	0	1				
HPMF dry, HPHF dry, HPMF wet	1	0				
HPHF dry and wet	4	3				

^1^Class I and II obesity defined as < 40% and > 40% overweight, based on analysis of body composition. ^2^Benjamini-Hochberg-adjusted P-values^68^ reported for categorical data are from either Chi squared or Fisher’s exact test; those for continuous data are from Mann Whitney tests. ^3^Effect size reported was Cohen’s V for tests involving categorical data and rank biserial for continuous data. The magnitude of the effect size is reported according to the rules of Cohen^69^ for Cramer’s V and Funder and Ozer^70^ for the rank biserial. ^4^95% CI 95% confidence interval. ^5^Body fat percentage determined by dual-energy X-ray absorptiometry. ^6^Ideal weight determined from body composition analysis from DXA, as described in the “Methods” Section. ^7^*HPMF* high protein medium fibre, *HPHF* high protein high fibre.

Significant values are in bold.

### Baseline data classified by degree of obesity

Of the 298 dogs, 118 (40%) and 180 (60%) were classified as having class I and class II obesity, respectively (Table 1). Not surprisingly given the classification criteria, body fat percentage (Mann–Whitney test, *P* < 0.001; rank biserial − 0.69 [very large effect]) and percentage overweight (Mann–Whitney test, *P* < 0.001; rank biserial − 1.00 [very large effect]) were both greater in dogs with class II obesity, but there were no differences between classes for age, sex, neuter status, therapeutic diet used and the presence of co-morbidities (*P* = 0.754 to 0.884, effect size 0.00 to 0.05 [very small or small effects]). Of the 116 cats enrolled, 68 (59%) and 48 (41%) were classified as having class I and class II obesity, respectively (Table 2). Again, body fat percentage (Mann–Whitney test, *P* < 0.001; rank biserial – 0.93 [very large effect]) and percentage overweight (Mann–Whitney test, *P* < 0.001; rank biserial − 1.00 [very large effect]) were greater in cats with class II obesity. Further, a greater proportion of cats with class II obesity were female (class I 24/68, 35%; class II 28/48, 58%; Chi-squared test, *P* = 0.037; Cramer’s V 0.21 [small effect]), but there were otherwise no differences in age, the presence of co-morbidities or diet between classes (Chi-squared tests, *P* = 0.435 to 0.979, effect size − 0.14 to 1.00 [small effects]).

### Prevalence of class I and II obesity over time

**Figure 1:**
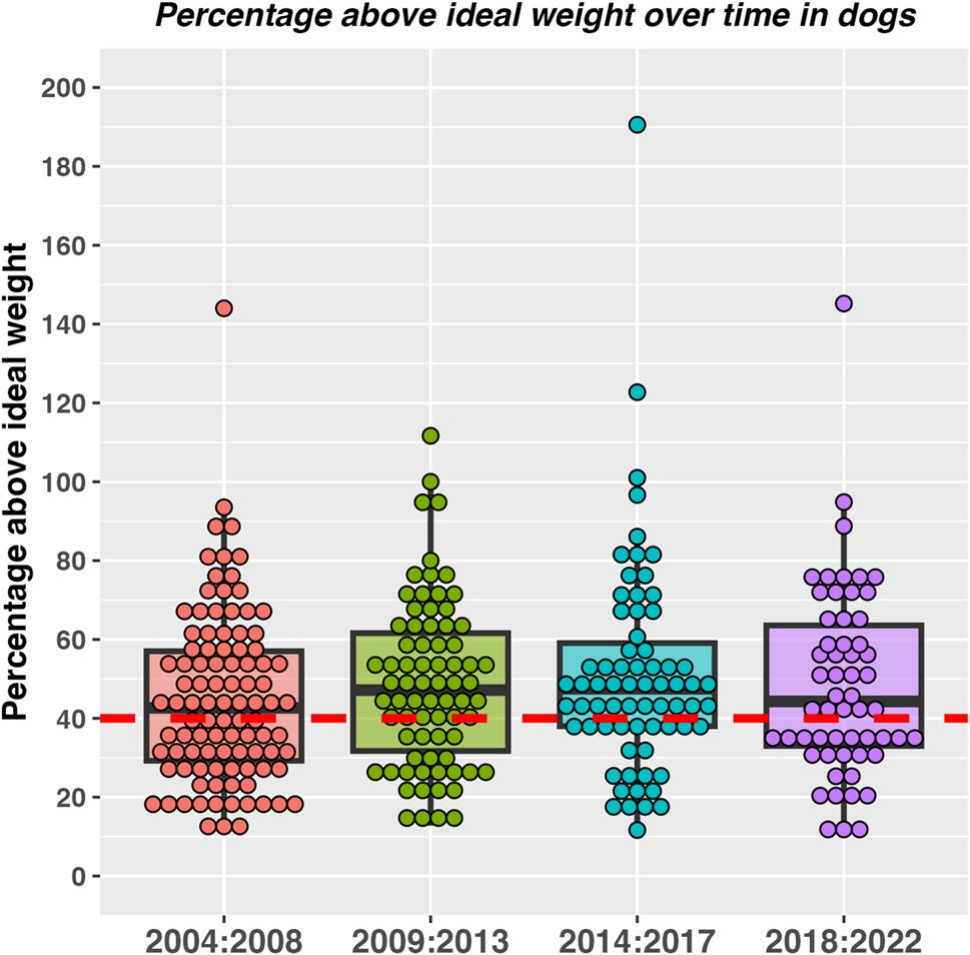
Percentage overweight in 298 dogs attending a specialist obesity care clinic stratified by year of enrolment (2004–2008, red; 2009–2013, green; 2014–2017, blue; 2018–2022, purple). The circles represent data from individual dogs or cats, thick horizontal black lines represent the median of each year group, whilst the upper and lower hinges of the boxes represent the inter-quartile range (IQR). The lower whisker represents the smallest observation greater than or equal to the lower hinge of the box minus 1.5 times the IQR; the upper whisker represents the largest observation less than the upper hinge of the box plus 1.5 times the IQR. The red dotted line depicts 40% overweight, meaning that animals below and above this line would be classified as classes I and II obesity, respectively. No time effect was evident for proportion of dogs in class I vs. class II obesity (Chi square test for trend, *P* = 0.358; Cramer’s V 0.07, 95% CI 0.00–1.00 [small effect]) and for the percentage overweight (Kruskal–Wallis test *P* = 0.490, η^2^ − 0.002, 95% CI − 0.01 to 0.04 [small effect]).

When assessed using a Chi-squared test for trend, the proportion of dogs in different classes did not change over time (2004–2008: 46 class I, 52 class II, 2009–2013: 25 class I, 49 class II; 2014–2017 23 class I, 46 class II; 2018–2022: 24 class I, 33 class II; *P* = 0.358; Cramer’s V 0.07, 95%-CI 0.00–1.00 [small effect]). Further, there was no difference in percentage overweight amongst dogs assigned to different year groups (Kruskal–Wallis test, *P* = 0.490; η^2^ − 0.002, 95% CI − 0.01 to 0.04 [small effect]; Fig. 1).

**Figure 2:**
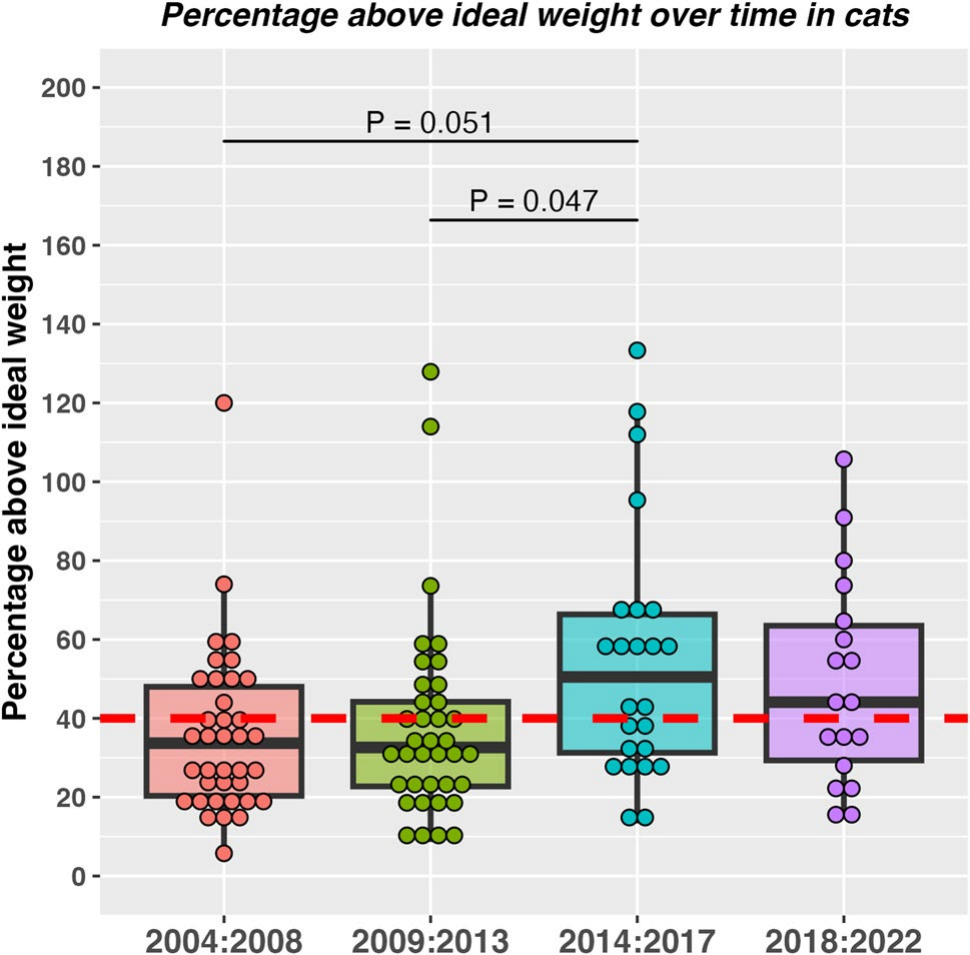
Percentage overweight in 116 cats (b) attending a specialist obesity care clinic stratified by year of enrolment (2004–2008, red; 2009–2013, green; 2014–2017, blue; 2018–2022, purple). The circles represent data from individual dogs or cats, thick horizontal black lines represent the median of each year group, whilst the upper and lower hinges of the boxes represent the inter-quartile range (IQR). The lower whisker represents the smallest observation greater than or equal to the lower hinge of the box minus 1.5 times the IQR; the upper whisker represents the largest observation less than the upper hinge of the box plus 1.5 times the IQR. The red dotted line depicts 40% overweight, meaning that animals below and above this line would be classified as classes I and II obesity, respectively. Significant time effects were evident both for the proportion in class I and II obesity (Chi square test for trend, *P* = 0.025, Cramer’s V 0.18, 95% CI 0.00–1.00 [small effect]) and percentage overweight (Kruskal–Wallis test *P* = 0.020, η^2^ 0.09, 95% CI 0.00 to 0.20 [medium effect]), with post-hoc testing indicating that the main group difference was between cats in year groups 2009–2013 and 2014–2017 (*P* = 0.047).

For cats, a significant time trend was evident amongst cats in different year groups (2004–2008: 25 class I, 12 class 2, 2009–2013: 25 class I, 12 class II; 2014–2017 10 class I, 14 class II; 2018–2022: 8 class I, 10 class II; Chi-squared test for trend, *P* = 0.025; Cramer’s V 0.18, 95% CI 0.00–1.00 [small effect]). Percentage overweight also differed amongst cats in the different year groups (Kruskal–Wallis test *P* = 0.020; η^2^ 0.09, 95% CI 0.00 to 0.20 [medium effect]; Fig. 2), with post-hoc testing indicating that the main group difference was between cats in year groups 2009–2013 and 2014–2017 (Dunn’s test, *P* = 0.047).

### Outcomes in animals with class I and class II obesity

Outcomes of controlled weight reduction for dogs in class I and II obesity are shown in Table 3. Compared with class I obesity dogs (78/118, 66%), fewer dogs with class II obesity (80/180, 44%) completed their weight reduction programme (Chi-squared test, *P* < 0.001; Cramer’s V 0.20 [small effect]). Further, dogs with class II obesity took longer to complete (Mann–Whitney test, *P* < 0.001, rank biserial -0.42 [very large effect]), likely because they lost a greater percentage of weight overall (Mann–Whitney test, *P* < 0.001; rank biserial -0.30 [medium effect]) at a slower average rate (Mann–Whitney test, *P* = 0.012; rank biserial 0.18 [small effect]). Dogs with class II obesity also attended more visits than dogs with class I obesity (Mann–Whitney test, *P* < 0.001; rank biserial -0.25 [medium effect]), but there were no differences in energy intake during weight loss, the number of times the diet was adjusted or the number of times weight loss stalled (Mann–Whitney tests, *P* = 0.217 to 0.460; rank biserial -0.05 to -0.09, very small effects; Table 3).

**Table 3:** Outcomes of weight loss in dogs with class I and class II obesity.

Variable	Obesity class^1^		*P* value^2^	Effect size^3^		
	I	II		Effect size	95% CI^4^	Interpretation
Number starting weight reduction	118	180	–	–	–	–
Number reaching target	78 (66%)	80 (44%)	** < 0.001**	**0.20**	0.10, 1.00	Small
Duration (days)	157 (0 to 953)	290 (0 to 1543)	** < 0.001**	** -0.42**	-0.52− 0.30	Very large
Percentage weight loss^5^	16.1 (-3.6 to 37.1)	23.0 (-2.6 to 47.9)	** < 0.001**	**-0.30**	-0.42 -0.17	Medium
Rate of weight loss (% per week)^5^	0.71 (-0.31 to 1.88)	0.48 (-0.26 to 2.29)	**0.012**	**0.18**	0.05, 0.31	Small
Energy intake during weight loss^6^						
kJ per kg^0.75^ per day	251 (171 to 377)	255 (169 to 365)	0.237	-0.09	-0.22, 0.05	Very small
kcal per kg^0.75^ per day	60.2 (42.3 to 90.2)	60.4 (40.3 to 87.2)	–	–	–	–
Number of visits	8 (1 to 30)	11 (1 to 50)	** < 0.001**	** -0.25**	-0.37, − 0.12	Medium
Number of diet adjustments	1 (0 to 10)	1 (0 to 13)	0.460	-0.05	-0.18, 0.09	Very small
Number of weight loss stalls	1 (0 to 12)	1 (0 to 22)	0.217	-0.09	-0.22, 0.05	Very small
Change in fat mass (%)^7^	-46.4 (-74.6 to -14.5)	-55.8 (-85.1 to -15.1)	** < 0.001**	**0.41**	0.24, 0.55	Very large
Change in lean mass (%)^7^	-3.6 (-20.0 to 13.2)	-10.6 (-23.9 to 4.7)	** < 0.001**	**0.61**	0.48, 0.71	Very large
Gained lean mass	21/73 (29%)	4/77 (5%)	** < 0.001**	**0.31**	0.16,1.00	Medium

Categorical data are expressed as number (%), whilst continuous data are expressed as median (range). ^1^Class I and II obesity defined as < 40% and > 40% overweight, based on analysis of body composition. ^2^Benjamini-Hochberg-adjusted P-values^68^ reported for categorical data are from either Chi squared or Fisher’s exact test; those for continuous data are from Mann Whitney tests. ^3^Effect size reported was Cohen’s V for tests involving categorical data and rank biserial for for continuous data. The magnitude of the effect size is reported according to the rules of Cohen^69^ for Cramer’s V and Funder and Ozer^70^ for the rank biserial. ^4^95% CI 95% confidence interval. ^5^Percentage weight loss and rate of weight loss expressed as a percentage of starting body weight. ^6^Average energy intake expressed as kJ or kcal per kg^0.75^ of ideal body weight in kg per day. ^7^Change in fat mass, change in lean mass and percentage of weight lost as fat were determined by comparing differences in body composition before and after weight reduction, as described in the “Methods” section. Note that changes in lean mass could be positive or negative, with positive values suggesting that lean tissue increased during the weight reduction period.

Significant values are in bold.

Outcomes of controlled weight reduction for cats in class I and II obesity are shown in Table 4. Like dogs, cats with class II obesity took longer to complete (Mann–Whitney test, *P* = 0.043; rank biserial -0.29 [medium effect]); however, whilst cats with class II obesity lost a greater percentage of weight overall (Mann–Whitney test, *P* = 0.048; rank biserial -0.27 [medium effect]), there was no difference in rate of weight loss between cats with class I and class II obesity (Mann–Whitney test, *P* = 0.126; rank biserial 0.21[medium effect]). Further, there were no differences in the number reaching target, the number of visits attended, the number of times the diet was adjusted and the number of weight loss stalls between cats in classes I and II obesity (Mann–Whitney tests, *P* = 0.118 to 0.586; rank biserial -0.06 to 0.21, very small to medium effects; Table 4).

**Table 4:** Outcomes of weight loss in cats with class I and class II obesity.

Variable	Obesity class^1^		*P* value^2^	Effect size^3^		
	I	II		Effect size	95% CI^4^	Interpretation
Number starting weight reduction	68	48				
Number reaching target	44 (65%)	22 (46%)	0.118	0.16	0.00, 1.00	Small
Duration (days)	194 (0 to 967)	350 (0 to 1548)	**0.043**	** -0.29**	**-**0.47,− 0.08	Medium
Percentage weight loss^5^	16.2 (**-**9.4 to 32.5)	27.2 (**-**3.7 to 41.1)	**0.048**	** -0.27**	**-**0.46, − 0.06	Medium
Rate of weight loss (% per week)^5^	0.54 (0.66 to 3.12)	0.34 (**-**0.06 to 1.33)	0.126	0.21	0.00, 0.40	Medium
Energy intake during weight loss^6^						
kJ per kg^0.67^ per day	218 (141 to 287)	224 (178 to 385)	0.586	**-**0.07	** -**0.29, 0.15	Very small
kcal per kg^0.67^ per day	52.0 (33.7 to 68.4)	53.6 (42.6 to 92.0)	–	–	–	–
Number of visits	8 (1 to 22)	10 (1 to 50)	0.512	**-**0.09	** -**0.29, 0.12	Very small
Number of diet adjustments	1 (0 to 7)	1 (0 to 24)	0.304	0.13	**-**0.08, 0.33	Small
Number of weight loss stalls	1 (0 to 11)	1 (0 to 28)	0.586	0.06	** -**0.16, 0.26	Very small
Change in fat mass (%)^7^	** -**58.0 (**-**86.2 to -29.9)	** − **69.8 (**− **83.3 to − 15.6)	0.135	0.29	**-**0.02, 0.55	Medium
Change in lean mass (%)^7^	**-**6.0 (1-5.9 to 6.0)	** -**10.2 (2-0.4 to -0.9)	**0.004**	**0.57**	0.31, 0.75	Very large
Gained lean mass	5/39 (13%)	0 /19 (0%)	0.253	0.17	0.00, 1.00	Small

Categorical data are expressed as number (%), whilst continuous data are expressed as median (range). ^1^Class I and II obesity defined as < 40% and > 40% overweight, based on analysis of body composition. ^2^Benjamini-Hochberg-adjusted P-values ^68^ reported for categorical data are from either Chi squared or Fisher’s exact test; those for continuous data are from Mann Whitney tests. ^3^Effect size reported was Cohen’s V for tests involving categorical data and rank biserial for continuous data. The magnitude of the effect size is reported according to the rules of Cohen ^69^ for Cramer’s V and Funder and Ozer ^70^ for the rank biserial. ^4^95% CI 95% confidence interval. ^5^Percentage weight loss and rate of weight loss expressed as a percentage of starting body weight. ^6^Average energy intake, expressed as kJ or kcal per kg^0.67^ of ideal body weight in kg per day. ^7^Change in fat mass, change in lean mass and percentage of weight lost as fat were determined by comparing differences in body composition before and after weight reduction, as described in the “Methods” section. Note that changes in lean mass could be positive or negative, with positive values suggesting that lean tissue increased during the weight reduction period.

Significant values are in bold.

### Changes in body composition in animals with class I and II obesity

Body composition data from both before and after weight reduction were available from 73/78 (94%) and 77/80 (96%) dogs with class I and II obesity, respectively, that completed their weight reduction protocol (Table 3). The magnitude of change in fat mass was greater in class II compared with class I dogs (Mann–Whitney test, *P* < 0.001; rank biserial 0.41 [very large effect]). The magnitude of lean mass change was also greater in class II compared with class I dogs (Mann–Whitney test, *P* < 0.001; rank biserial 0.61 [very large effect]), whilst a greater proportion of class I obesity dogs gained lean tissue (Chi-squared test, *P* < 0.001; Cramer’s V 0.31 [medium effect]) compared with class II obesity dogs. It was possible that the dogs with class II obesity had lost more lean tissue simply because their percentage weight loss was greater. To examine this, a multiple linear regression model was constructed to determine the association between lean tissue change and obesity class, whilst accounting for the percentage of weight lost (Table 5; dog model). In this model, both class (regression coefficient -3.349, *P* = 0.007) and percentage weight loss (regression coefficient -0.394, *P* = 0.007) were independently associated with change in lean tissue mass.

**Table 5:** Multiple linear regression assessing the association between change in lean tissue mass and both obesity class and percentage weight in dogs with obesity undergoing controlled weight reduction.

Parameter	Estimate^a^	95% CI^a^	Adjusted R^2b^	*P* value
Dog model	–	–	0.394	**< 0.001**
Predictor variables				
Obesity class	** -**3.349	** -**5.761, -0.937	–	**0.007**
Weight loss (per %)	** -**0.394	** -**0.533, -0.255	–	** < 0.001**
Cat model	–	–	0.301	** < 0.001**
Predictor variables				
Obesity class	** -**1.542	** -**5.259, 2.175	–	0.409
Weight loss (per %)	** -**0.349	** -**0.569, -0.129	–	**0.002**

^a^Estimate and 95% confidence interval (95% CI) of the regression coefficient for the predictor variable; for obesity class, the coefficient represents the expected difference in percentage lean mass change for dogs with class II compared with class I obesity dogs; for percentage weight loss, the estimate represents the change in lean mass for each percentage of weight lost. ^b^Model performance assessed by calculating R^2^ adjusted for the number of predictors in the model.  Significant values are in bold.

Body composition data from both before and after weight reduction were available from 39/44 (89%) and 19/22 (86%) cats with class I and II obesity, respectively, that completed their weight reduction protocol (Table 4). The magnitude of change in lean mass was greater (Mann–Whitney test, *P* < 0.001; rank biserial 0.57 [very large effect]) in cats with class II compared with class I obesity. However, there were no differences between classes in either change in fat mass (Mann–Whitney test, *P* = 0.135; rank biserial 0.29 [medium effect]) or the proportion of cats that gained lean tissue (Chi-squared test, *P* = 0.253; Cramer’s V 0.17 [small effect]). As with dogs a multiple linear regression model was constructed to determine the association between lean tissue change and obesity class, whilst accounting for the percentage of weight lost (Table 5; Cat model). In this model, an association was identified between lean tissue change and percentage weight loss (regression coefficient -0.349, *P* = 0.002) but not with obesity class (regression coefficient -1.542, *P* = 0.409).

## Discussion

The primary aim of the current study was to assign dogs and cats with overweight and obesity into two classes (I; ≤ 40% overweight; II > 40% overweight) based on body composition analysis by DXA, and then to compare differences in weight loss outcomes between classes. A further aim was to determine whether the proportion of dogs and cats with different obesity classes has changed over time, based on referrals to a specialist obesity care clinic. Overall, approximately half of all dogs and cats seen were classified as having class II obesity and, during controlled weight reduction, they lost weight more slowly and lost more lean tissue mass, than those with class I obesity.

In human medicine, obesity is subdivided into three classes (I, II and III) depending on degree of adiposity as defined by BMI^17^. As mentioned above, morbidity and mortality risk differ amongst classes^22^, with individuals having class III obesity being at greatest risk^23–27^. The use of classes, rather than historical descriptions (such as ‘severe’ or ‘morbid’), also avoids the use of stigmatising language, not least given the prevalence and negative consequences of weight stigma both in society^28^ and amongst medical professionals^29^. In the current study, the cut-point between the two classes (40% overweight) was chosen because this value signifies the upper limit of the 9-point BCS in dogs and cats^19,20^. Given this difference in assigning the cut-point, the two canine and feline obesity classes are not directly comparable with human classes of obesity, although it does then provide a similar opportunity to explore differences in health consequences and outcomes.

To examine possible associations with morbidity, we compared differences in comorbidities between obesity classes. In contrast to humans, the proportion of individuals that had at least one comorbidity did not differ between animals with class I and class II obesity. However, we should be cautious in interpreting these results. Firstly, because the study was undertaken at a specialist obesity care clinic, the cases studied might not have been representative of pet dogs and cats attending primary care practices. This might explain why most animals studied had at least one comorbidity, with the effect that the statistical comparison was probably not meaningful. Further, many different comorbidities were present which were diverse in causes and consequences (Supplementary Data S1); grouping these comorbidities for the purpose of statistical analysis might have meant that genuine associations between obesity class and single comorbidities were missed. This was partially addressed in dogs by assessing orthopaedic, cardiorespiratory and dermatological disease separately; however, different diseases would still have been grouped into these single body system categories. Therefore, associations between obesity class and the presence of comorbidities would be better studied in epidemiological studies using larger, and more representative populations. A further limitation was the fact that we did not assess associations between obesity class and other adverse health impacts known to be associated with overweight status, such as lifespan, quality of life and comorbidities^2–10^. Again, therefore, further studies would be required to assess the full impact on of obesity class on health and well-being.

In humans, outcomes of conventional weight loss interventions are similar amongst classes, with individuals typically losing ~ 5–6% of their starting weight, whatever their obesity class^30^. In contrast, some weight loss outcomes did differ between obesity classes in dogs and cats of the current study: although both cats and dogs with class II obesity lost more weight than those in class I, weight reduction protocols took longer overall. Further, the rate of weight loss was slower, and more visits were required in dogs, but not cats, with class II obesity. Longer protocols requiring more visits are likely to be more challenging for owners, given the time commitment required, whilst a slower rate of weight loss could also be discouraging, increasing the chances of non-compliance or early discontinuation. This might explain why, compared with class I obesity, a lesser proportion of dogs with obesity class II reached their target weight. The slower rates of weight loss and poorer compliance are likely to be the result of the known physiological adaptations that occur during weight reduction, which antagonise the progress and can also promote weight regain^31^. In humans, several mechanisms have been identified including alterations in circulating concentrations of appetite-related hormones (e.g. increased ghrelin and gastric inhibitory polypeptide; decreased leptin, peptide YY, cholecystokinin, amylin, insulin and glucagon-like peptide-1)^31^, compensatory changes to energy homoeostasis (decreased energy expenditure due to reduced body mass and enhanced metabolic efficiency)^32^, altered nutrient metabolism that can alter energy homeostasis^33^) and subjective changes in appetite (e.g. increased perception of hunger^31^). Although research is more limited, some of these factors have also been demonstrated to occur during weight loss in companion animals. For example, weight loss in dogs leads to an increase in ghrelin concentration and a decrease in leptin concentration^34^. Further, energy expenditure decreases during weight reduction in both cats^35^and dogs^36^ with obesity, and maintenance energy requirements remain low even during subsequent weight maintenance^37,38^.

Whatever the underlying mechanisms, given that outcomes are worse in dogs and cats with class II obesity, future research should aim to develop treatments and strategies better tailored to such individuals. For example, therapeutic diets could be better formulated to mitigate lean tissue loss, perhaps, by altering protein and amino acid profiles or adding functional ingredients; in this respect, l-carnitine has effects on protein turnover and energy expenditure^39^, and is already included in therapeutic weight loss diets given its positive effects in promoting muscle mass^40^. Pharmaceutical agents could also be considered; licensed drugs were previously available for dogs, such as dirlotapide, which produced significant weight reduction in clinical trials^41^ but had side effects, and was eventually withdrawn from the market. Alternatively, off-label use of the newer human obesity drugs could be considered, such as semaglutide, which produces significant weight loss and also improves health outcomes such as reducing cardiometabolic risk and increasing physical function^42^. If such drugs were similarly effective in cats and dogs, there would be more therapeutic options, not least in cases where progress on a therapeutic weight loss diet is slow. Until either novel diets or drugs become available, a pragmatic approach could instead be considered for dogs and cats with class II obesity, such as using partial weight reduction protocols^43^. With such protocols, the target weight set is deliberately greater than the ideal weight, with the intention of maximising any benefits, such as functional improvements and quality of life whilst, concurrently, reducing the chances of failure because of non-compliance and discontinuation^44^. Partial weight reduction protocols are more likely to succeed than complete protocols^43^, but are a compromise and some negative health consequences might remain; for example, given that animals remain in overweight condition even after the end of their protocol, there is still likely some increased risk of developing comorbidities^8–10^.

Despite the longer duration and greater number of visits, there were no significant differences between classes in the number of times the weight loss stalled or the number of times that a change in the diet plan was required. The number of weight loss stalls is a crude metric for the challenges faced by an owner during a weight loss plan because more weight loss stalls would be expected in more challenging plans. Similarly, poor compliance is likely when owners are finding the weight reduction process challenging and, in such cases, more diet changes might be expected. Therefore, the current results might suggest that, despite a longer and slower process, the day-to-day challenges were not different for owners. Arguably, however, these variables do not capture the full extent of challenges experienced by owners whose pet is undergoing controlled weight reduction. To explore this more completely, additional metrics would be needed including the amount of food-seeking behaviour and diary records of non-compliance with the diet. Further, since this was an observational study, causality cannot be assumed and, in fact, there might be inverse causality. In this respect, owners who are finding the process more challenging might be more resistant to changing the plan, for example, by reducing the daily food portion; this might then lead to a slower rate of weight loss, with the effect being a longer plan overall.

Compared with dogs with class I obesity, those with class II obesity lost a greater amount of fat during their controlled weight reduction plan which is, perhaps, not surprising given that their starting fat mass was greater. However, change in fat mass during weight reduction did not differ between cats in the two obesity classes. This finding might be explained by the variability in how much body fat mass changed and the fact that there was marked overlap between classes (class I -86 to -30%; class II -83% to -16%). Therefore, although a medium effect size was observed, the group sizes might have been too small to enable the detection of a statistically-significant difference between group medians in hypothesis testing. To address this, further work would be required where body composition changes during weight reduction are assessed in a larger population of cats with obesity.

Loss of lean tissue during controlled weight reduction has also previously been reported in humans^45,46^, cats and dogs^13,14,44^. A novel finding of the current study was that losses were greater in dogs with class II compared with class I obesity. Since previous studies have shown that lean tissue loss correlates with overall percentage weight loss^13,14,44^, this class difference might simply be due to those with class II obesity animals having lost more weight. Indeed, in cats, the class difference disappeared when percentage weight loss was accounted for using multiple regression. However, in dogs, the class effect on lean tissue loss remained even after adjusting for percentage weight loss. Possible mechanisms for the additional lean tissue loss seen in dogs with class II obesity include a difference in severity of insulin resistance along with differences in adipokine profiles. For example, in experimental murine studies of obesity-related insulin resistance, an increased loss of muscle mass occurs via insulin receptor substrate-1/phosphatidylinositol-3-kinase Akt pathway down-regulation^47^; since adiponectin inhibits the muscle degradation that occurs via this pathway^48^, the decreased adiponectin concentrations that arise in individuals with obesity might be contributory. These results are interesting given that, in cats, pre-weight-loss adiponectin concentrations are negatively correlated with the amount of lean tissue lost during weight reduction^49^, although this has not been seen in dogs. Of course, differences in other adipokines might also be responsible, such as resistin, which has been implicated in impairment of myogenesis and maintenance of muscle mass in humans^50^. It was beyond the scope of the current study to explore differences in insulin resistance and adipokine patterns between the obesity classes, but this could be considered in a future study.

Whether the additional lean tissue lost in animals with class II obesity is the result of the overall percentage of weight lost (both dogs and cats) or other factors (dogs only), it is an important consideration; for example, loss of muscle mass is an independent predictor of mortality in human chronic diseases^48^, and whole-body protein catabolism is increased both in humans with type 2 diabetes mellitus and those with obesity^47^. As mentioned above, lean tissue mass decreases during weight loss using diet-based strategies in humans^45^, and the consequences of this have been described, including decreased metabolic rate and increased risk of injury^46,51,52^. A decrease in metabolic rate can make any weight loss hard to sustain, and this might be a reason for subsequent regain of weight^46^. Given the adverse effects seen in people, it is likely that excessive lean tissue loss during weight reduction in dogs and cats might have similar negative effects. For this reason, the authors recommend taking steps to limit lean tissue loss during controlled weight reduction, such as using partial weight reduction protocols. In a recent study in cats with obesity, such an approach limited the amount of lean tissue lost^43^.

Approximately half of all the animals in this study (60% dogs, 41% cats) had class II obesity, with the proportion remaining relatively stable over time in dogs but not cats, where the prevalence between 2014 and 2017 was greater than the prevalence between 2009 and 2013. Given that these cases were seen at a specialist obesity care clinic, it is unclear as to whether a similar prevalence occurs in the wider pet population. Nonetheless, the findings are important because their visual and physical characteristics are not appropriately depicted by the commonly used 9-point BCS system^19,20^. The current study used DXA to classify such cases, a technique that is not widely available, thereby limiting the generalisability of the current results to primary care practice. Even with investment in such equipment, the additional cost implications for owners of pets with obesity might limit its use. Therefore, for the concept of obesity class to be translated to primary care veterinary practice, simple clinical assessments would be required. One option might be to use a different approach to assess adiposity, such as the body fat index (BFI)^17,18^. With this metric, visual and physical characteristics are subjectively assessed using a rubric, which enables a crude estimate of body fat mass to be made. In this respect, BFI scores of 30, 40, 50, 60 and 70 correlated with ~ 14%, ~ 30%, ~ 67%, ~ 100%, and ~ 167% overweight. Therefore, animals with a BFI of either 30 or 40 could be classified as obesity class I, whilst those with a BFI of 50, 60 or 70 could be classified as obesity class II. One downside of the BFI is that it is less well known by veterinary professionals compared with the 9-point BCS. Further, the visual and physical characteristics assessed differ in the systems used for dogs and cats, making the system more complicated than BCS. A second option for a clinical tool to estimate body fat mass is to use zoometry, again as previously described^17,18^. However, this technique is complicated, taking longer to complete and requiring greater patient co-operation, whilst marked variability can occur with tape measure measurements, adversely affecting accuracy^53^, not least when used in multi-veterinarian practices or in more challenging animals (aggressive or nervous). Such issues limit widespread acceptance of alternative techniques to the 9-point BCS, not least in busy primary care practices where the available time in consultations might be limited.

Therefore, the third option would be to consider redesigning the existing 9-point BCS system, for example, by adding more categories for cats and dogs that are > 40% overweight. This would require further research whereby visual and physical characteristics of dogs and cats with class II obesity could be assessed to determine characteristics distinguishing these animals from those of existing classes (especially BCS 9). Any revised system would then need to be validated by comparing its performance with a gold-standard measure of body fat mass such as DXA. Of course, as with any new system, there might then be challenges with acceptance from veterinary professionals. A pragmatic approach could be to continue to use the existing BCS system, but also flag any individuals whose visual and palpable characteristics suggest that they are beyond the upper limit of the scale (i.e., by recording them as “9+ ” or “above 9”). One strength of the current 9-point BCS is that it can be used to estimate ideal weight^54^, because the relationship between body fat mass and BCS is approximately linear^19,20,55^. A limitation of such the pragmatic approach of adding a “9+ ” category, is that estimates of ideal weight in that category would be problematic. For such cases, other methods would be required to estimate ideal weight, for example, by assessing historical weight records to identify a prior adult weight where the dog was in optimal body condition, which can then be used as an ideal weight.

As for any scientific research, there are limitations that warrant consideration, some of which have already been discussed, including the fact that the study population might not be representative of the general pet population. Owners who agree to be referred to such a clinic might well be more motivated and, as a result, outcomes might be more favourable than for animals in the general pet population. Further, there was variability among animals and the study population was relatively small (especially in cats), and this might have obscured the identification of relationships between the degree of obesity and outcomes of weight reduction. This was compounded by the fact that some data were missing, for example, post-weight-reduction body composition data in animals where DXA was not performed after weight reduction. This limited our ability to analyse changes in body composition during the weight reduction period and might have contributed to the failure to detect a significant difference in fat mass change between classes in cats despite a medium effect size.

In conclusion, the subclassification of canine and feline obesity into classes I and II has been described, with animals with class II obesity having worse weight outcomes than those with class I obesity. Based on such a classification, many pet dogs and cats presenting to a specialist obesity care clinic would have class II obesity and, therefore, not be well represented by the current 9-point BCS system.

## Methods

### Animals

All participating animals had been referred to a *specialist obesity care clinic* for dogs and cats (Royal Canin Weight Management Clinic, University of Liverpool, Neston, UK) for investigation and management of obesity or obesity-related disorders between October 2004 and December 2022. To be eligible, animals had to have had body composition measured by DXA and had to have reached an endpoint for their weight reduction protocol, in a similar manner to previous studies in cats and dogs^15,16^. Animals that reached target weight were classified as “completed,” whilst those that did not were classified as “stopped prematurely” (including euthanasia or stopping at the owner’s request). Animals whose weight reduction programme was still ongoing in December 2022 were not eligible for inclusion. Finally, animals had to be above their ideal weight (based on body composition analysis, see below), could not have a comorbidity that would make weight loss contraindicated, and controlled weight reduction would reasonably be expected to improve their health.

The University of Liverpool Veterinary Research Ethics Committee (RETH000353 and VREC793) and the Royal Canin Ethical Review Committee (150720-55) both approved the study protocol. As part of the University approval, the nature of the procedures performed were considered and, specifically, whether they should be classified as experimental procedures. In this respect, all clinical procedures were conducted in accordance with relevant guidelines (e.g. standard operating procedures) and regulations. Further, the foods used were fed for the clinical benefit of the study animals, and were commercially-available therapeutic diets commonly used by veterinarians to manage obesity. As a result, in the ethical approvals granted (RETH000353 and VREC793), neither the clinical procedures used nor the clinical use of the therapeutic diets were deemed to involve animal experimentation and, therefore, fell outside the remit of national legislation (e.g. the revised Animals [Scientific Procedures] Act 1986). The study is reported in accordance with ARRIVE guidelines (https://arriveguidelines.org).

### Measurement of body weight and body composition

Body weight was measured by electronic weigh scales, which were regularly calibrated using test weights (2–50 kg; guaranteed to be accurate to within ≤ 0.5%; Blake and Boughton Ltd., Thetford, UK). Body composition was analysed in all patients using the same fan-beam DXA (Lunar Prodigy Advance; GE Lunar; Madison, USA), calibrated on a weekly basis using a phantom supplied by the company, and which has previously been shown to have high precision for repeat analysis in dogs^12^. Patients were either sedated (if DXA alone was performed) or anaesthetised if required for additional procedures, and scanned in dorsal recumbency, as previously described^13,14^. Purpose-designed computer software (Encore 2004, 8.70.005; GE Lunar) was used for data analysis^13,14^.

### Weight reduction protocol

Full details of the weight reduction protocol used have been published previously^13–16^. Briefly, at the first visit, patients were weighed, their BCS assessed and their body composition measured by DXA (see below). Health status was determined by routine haematology, serum biochemistry, free thyroxine measurement (in dogs) and urinalysis. If necessary, additional diagnostic investigations (e.g. diagnostic imaging, additional laboratory investigations) were performed to determine the status of any comorbidities. A tailored weight reduction protocol was then formulated for each animal, again as previously described^13,14^. Briefly, animals were fed dry or moist therapeutic diets appropriate for their species (Tables 6 and 7; Royal Canin, Aimargues, France), which were either high in both protein and fibre (HPHF) or were high in high protein with a moderate fibre content (HPMF). The diet choice depended on owner and animal preferences.

**Table 6:** Average composition of the therapeutic diets used for weight reduction in 298 dogs with obesity.

Criterion	HPMF dry^1^		HPHF dry^2^		HPMF wet^3^		HPHF wet^4^	
ME content	3275 kcal per 1000 g		2900 kcal per 1000 g		563 kcal per 1000 g		602 kcal per 1000 g	

	As fed^5^	Per 1000 kcal	As fed^5^	Per 1000 kcal	As fed^5^	Per 1000 kcal	As fed^5^	Per 1000 kcal
Moisture	10	29	10	33	86	1528	83	1379
Crude protein	34	105	30	105	7	128	8.5	141
Crude fat	10	31	10	33	2	37	2.0	33
Crude fibre	8	24	17	58	1	18	2.0	53
Total dietary fibre	18	56	28	97	1.4	26	3.2	33
Ash	8	24	6	20	1.5	27	1.5	25

*DM* dry matter, *ME* metabolisable energy content, calculated using the National Research Council 2006 predictive equation based on total dietary fibre (TDF).

^1^Obesity management, Royal Canin, ^2^Satiety weight management, Royal Canin, ^3^Obesity management, Royal Canin, ^4^Satiety weight management, Royal Canin, ^5^Expressed as grams per 100 g.

**Table 7:** Average composition of the therapeutic diets used for weight reduction in 116 cats with obesity.

Criterion	HPMF wet^1^		HPHF wet 2^2^		HPMF dry 1^3^		HPHF dry 2^4^	
ME content	3394 kcal/kg		2963 kcal/kg		620 kcal/kg		677 kcal/kg	

	As fed^5^	Per 1000 kcal	As fed^5^	Per 1000 kcal	As fed^5^	Per 1000 kcal	As fed^5^	Per 1000 kcal
Moisture	9.9	29	9.8	33	83.7	1352	83.9	1237
Crude protein	40.2	118	32.6	110	7.6	120	7.4	112
Crude fat	9.7	29	8.7	29	1.9	31	2.3	33
Crude fibre	6.3	19	13.5	46	1.4	23	1.2	17
Total dietary fibre	13.0	38	21.6	73	1.6	26	1.4	21
Ash	7.6	22	8.1	27	1.6	25	1.7	25

*DM* dry matter, *ME* metabolisable energy content, calculated using the National Research Council 2006 predictive equation based on total dietary fibre (TDF).

^1^Obesity management, Royal Canin, ^2^Satiety weight management, Royal Canin, ^3^Obesity management, Royal Canin, ^4^Satiety weight management, Royal Canin, ^5^Expressed as grams per 100 g.

In dogs, the initial food allocation for the weight reduction protocol was determined based on ideal weight (as calculated by DXA), in a two-stage process; first, the metabolisable energy requirement for maintenance (a.k.a. maintenance energy requirement, MER) was estimated as 440 kJ (105 kcal) × body weight [kg]^0.75^/day; the degree of restriction for each dog was then individualised based upon sex (female < male), neuter status (neutered < intact) and other factors (i.e. presence of associated diseases that might affect energy expenditure such as orthopaedic disease). This initial food allocation was typically between 50 and 65% of MER at ideal weight (as determined by DXA, see below). In cats, the initial energy allocation for the weight reduction protocol was 126–146 kJ (30–35 kcal) per kg of ideal weight (again, as determined by DXA, see below), with no adjustment for sex, neuter status or the presence of comorbidities.

In addition to advice about feeding the therapeutic diet, owners also received tailored advice on lifestyle alterations to assist the weight reduction process. This included a physical activity plan, tailored to owner circumstances, individual animal factors and the presence of comorbidities. Advice could include recommendations about play activity in both dogs and cats, but also walking, running, agility training and hydrotherapy, in dogs.

After the initial visit, animals were then reassessed every 7 to 21 days to have their body weight measurements taken, and changes were made to the dietary and exercise plan if necessary. In animals that reached their target weight, a final evaluation was conducted. Health status was determined based on physical examination, haematology, serum biochemical analysis and urinalysis. Body weight and body condition were recorded, and body composition was reassessed by DXA.

### Determining ideal body weight and obesity class

For the purposes of this study, ‘ideal weight’ was defined as the weight at which body fat mass was determined to be optimal, assumed to be ~ 15–25% for cats^13,18^ and 20–30% for dogs^12,14,17^. To estimate ideal weight, DXA measurements of lean mass, fat mass and bone mineral content (in grams), before weight reduction, were entered into a spreadsheet (Excel for Mac, version 16.71, Microsoft). This spreadsheet incorporated a bespoke mathematical formula for modelling body composition changes during weight loss^13,14^, which assumed a median proportional loss in tissue mass of 83% fat: 17% lean; the ratio used was based on analysis of all available body composition data from the *specialist obesity care clinic* using the same diets, same DXA machine and software. Once ideal weight had been determined, percentage overweight was then calculated using the following formula:

$$Percentage \,\,overweight= \left\{\left(start \,\,weight \left[kg\right]-ideal \,\,weight [kg]\right)\div \left(ideal \,\,weight \left[kg\right]\right)\right\}\times 100.$$

Animals were then assigned to one of two obesity classes: class I (< 40% overweight) and class II (≥ 40% overweight). This cut-point was selected because it equates to BCS 9 in both cats and dogs^19,20,55,56^.

### Determining changes in body composition during controlled weight reduction

Where body composition data were available from both before and after weight management (i.e. in most dogs and cats that reached their target weight), changes in body composition were assessed. Changes in fat and lean mass were determined from the following equation:

$$Change\,\, in \,\,mass \left(\%\right)= \left\{\left({mass}_{pre} \left[g\right]-{mass}_{post} [g]\right)\div \left({mass}_{pre} \left[g\right]\right)\right\}\times 100.$$

Changes in fat mass were always negative, suggesting that adipose tissue was always lost during weight reduction; however, changes in lean mass could be positive or negative: a positive lean mass change was seen when the lean mass after weight reduction was greater than that before, and suggested some gain of lean tissue.

### Determining sample size

Since the study was observational, and there have been no previous attempts to classify canine and feline obesity according to class, a sample size calculation was not possible. Instead, we aimed to include as many dogs and cats as possible from those that had attended the obesity care clinic during the timeframe. However, the numbers of both dogs and cats in the current study was greater than the numbers studied in previous publications from the same obesity care clinic^3,13–16,43,56^.

### Data handling and statistical analysis

#### Dataset, variables assessed and missing data

The dataset on which all statistical analyses were conducted is provided in the online supplementary information for the study (Supplementary Data S1). Continuous data are expressed as median and range, whilst categorical data are reported as a number (percentage). In addition to obesity class, baseline variables recorded were age, breed, sex, neuter status, comorbidities (classified according to body system affected), body fat percentage, the percentage overweight and diet used (classified according to the macronutrient profile). Further, to assess changes in the distribution of obesity classes over time, animals were assigned to one of four different year groups, covering the period of enrolment to the study: 2004–2008, 2009–2013, 2014–2017 and 2018–2022. Outcome variables recorded were number reaching target weight, duration, percentage weight loss, rate of weight loss, average energy intake during weight loss, number of visits, number of times the diet was adjusted (e.g. by changing the daily food portion or type of diet used), number of weight loss stalls (where there had either been no weight loss or weight gain since the previous visit), percentage change in fat mass, percentage change in lean mass and number gaining lean mass.

Post-weight-reduction body composition data from unavailable from 140 dogs and 50 cats that did not complete their weight reduction protocols, and a further 8 dogs and 8 cats that did complete, because follow-up DXA was not performed. Further, average energy intake data could not be calculated in 9 dogs and 8 cats that did not return after the first visit. Otherwise, there were no missing data for any other variable assessed (Supplementary Data S1).

#### Statistical software

Statistical analysis was performed using an online open-access statistical language and environment (R, version 4.2.3)^57^ with several additional packages: ‘car’ version 3.1.2^58^, ‘dplyr’ version 1.1.2^59^, ‘effectsize’ version 0.8.3^60^, ‘FSA’ version 0.9.4^61^, ‘ggplot2’ version 3.4.2^62^, ‘ggstatsplot’ version 0.11.1^63^, ‘lmtest’ version 0.9.40^64^, ‘MASS’ version 7.3.60^65^, ‘psych’ version 2.2.9^66^ and ‘rstatix’ version 0.7.1^67^. Except where indicated, two-sided *P*-values were adjusted to control the false-discovery rate using the Benjamini–Hochberg method^68^, with the level of significance being *P* < 0.05.

#### Statistical analyses

The distributions of all continuous data were first assessed by examining histograms, Q-Q plots and using the Shapiro–Wilk Test. Given that most datasets did not have a normal distribution, a decision was made to use non-parametric statistical methods. A Chi-squared test for trend was used to establish if there was any change in the prevalence of class I and class II obesity over time (e.g. across the four year groups), with the effect size determined by calculating Cramer’s V alongside its 95% confidence interval (95% CI); these effect sizes were interpreted according to the rules of Cohen^69^ with 3 degrees of freedom; therefore, values of 0.06, 0.17 and 0.29 denoted small, medium and large effects, respectively. The Kruskal–Wallis test was used to explore changes in percentage overweight over time further, by comparing animals in the four year groups. Post-hoc comparisons amongst groups were made using Dunn’s test, with the false-discovery rate controlled using the Benjamini–Hochberg adjustment^68^. The effect size was estimated by calculating η^2^ alongside its 95%-CI, whilst the magnitude of the effect was interpreted according to the rules of Cohen^69^, with values of 0.01, 0.06 and 0.14 denoting small, medium and large effect sizes, respectively.

The Mann–Whitney test was used to compare continuous baseline and outcome variables between animals with class I and class II obesity, with the effect size determined by calculating the rank biserial and its 95% confidence interval (95% CI). Rank biserial effect sizes were interpreted according to the rules of Funder and Ozer^70^, with larger values (positive or negative) indicating larger differences between groups (tiny: < 0.05: very small: 0.05–0.10; small: 0.10–0.20; medium: 0.20–0.30; large: 0.30–0.40; very large: > 0.40). Finally, either the Chi-squared test or Fisher’s exact test (if one or more cells in the contingency table had an expected count of < 5) were used to assess categorical baseline and outcome variables between animals with class I and class II obesity. Once again, Cramer’s V was used to indicate effect size and interpreted according to Cohen^69^, albeit with 1 degree of freedom; therefore, values of 0.10, 0.30 and 0.50 denoted small, medium and large effects, respectively. For diet, two comparisons were made: cats fed dry food exclusively with those fed either wet food exclusively, or a combination of wet and dry food; sex and neuter status were compared separately in dogs, but only sex was compared in cats (given that all were neutered). When assessing comorbidities in dogs, the presence of any comorbidity was assessed, as well as separate assessments for orthopaedic, cardiorespiratory or dermatological disease (the three most common body systems affected); given the smaller number of cats, comparisons were only made for the presence or absence of any comorbidity and presence or cardiorespiratory disease. No statistical comparisons amongst breeds were made because dogs were from many different breeds and the numbers within each breed were often small (Tables 1 and 2).

Finally, multiple linear regression was used to examine associations between change in lean mass and obesity class, whilst accounting for percentage weight loss. Model performance was assessed using adjusted R^2^ and the associated *P* values, whilst several methods were used to ensure that model assumptions were met: first, normality of residuals was confirmed by visually-inspecting Q-Q plots and using the Shapiro–Wilk test; second, homogeneity of variance was confirmed using visual inspection of a plot of fitted values against the square root of the standardized residuals, and also with the Breusch-Pagan test; third, no influential datapoints were identified using Cook’s distance; finally, absence of multicollinearity was confirmed by assessing variance inflation factors (VIF) and ensuring that all were < 4.

### Supplementary Information

Dataset S1.

## Data Availability

All data generated or analysed during this study are included in this published article and its supplementary information files (e.g. Supplementary Data S1).
